# The value of machine learning based radiomics model in preoperative detection of perineural invasion in gastric cancer: a two-center study

**DOI:** 10.3389/fonc.2023.1205163

**Published:** 2023-06-14

**Authors:** Xujie Gao, Jingli Cui, Lingwei Wang, Qiuyan Wang, Tingting Ma, Jilong Yang, Zhaoxiang Ye

**Affiliations:** ^1^ Department of Radiology, Tianjin Medical University Cancer Institute and Hospital, Tianjin, China; ^2^ Department of Radiology, National Clinical Research Center for Cancer, Tianjin, China; ^3^ Department of Radiology, Tianjin’s Clinical Research Center for Cancer, Tianjin, China; ^4^ The Key Laboratory of Cancer Prevention and Therapy, Tianjin, China; ^5^ Department of General Surgery, Weifang People’s Hospital, Weifang, Shandong, China; ^6^ Department of Radiology, Weifang People’s Hospital, Weifang, Shandong, China; ^7^ Department of Radiology, Tianjin Cancer Hospital Airport Hospital, Tianjin, China; ^8^ Department of Bone and Soft Tissue Tumor, Tianjin Medical University Cancer Institute and Hospital, Tianjin, China

**Keywords:** radiomics, perineural invasion, gastric cancer, computed tomography, nomogram

## Abstract

**Purpose:**

To establish and validate a machine learning based radiomics model for detection of perineural invasion (PNI) in gastric cancer (GC).

**Methods:**

This retrospective study included a total of 955 patients with GC selected from two centers; they were separated into training (n=603), internal testing (n=259), and external testing (n=93) sets. Radiomic features were derived from three phases of contrast-enhanced computed tomography (CECT) scan images. Seven machine learning (ML) algorithms including least absolute shrinkage and selection operator (LASSO), naïve Bayes (NB), k-nearest neighbor (KNN), decision tree (DT), logistic regression (LR), random forest (RF), eXtreme gradient boosting (XGBoost) and support vector machine (SVM) were trained for development of optimal radiomics signature. A combined model was constructed by aggregating the radiomic signatures and important clinicopathological characteristics. The predictive ability of the radiomic model was then assessed with receiver operating characteristic (ROC) and calibration curve analyses in all three sets.

**Results:**

The PNI rates for the training, internal testing, and external testing sets were 22.1, 22.8, and 36.6%, respectively. LASSO algorithm was selected for signature establishment. The radiomics signature, consisting of 8 robust features, revealed good discrimination accuracy for the PNI in all three sets (training set: AUC = 0.86; internal testing set: AUC = 0.82; external testing set: AUC = 0.78). The risk of PNI was significantly associated with higher radiomics scores. A combined model that integrated radiomics and T stage demonstrated enhanced accuracy and excellent calibration in all three sets (training set: AUC = 0.89; internal testing set: AUC = 0.84; external testing set: AUC = 0.82).

**Conclusion:**

The suggested radiomics model exhibited satisfactory prediction performance for the PNI in GC.

## Introduction

Perineural invasion (PNI) is a common mechanism of malignant nerve invasion. Although the exact mechanisms of metastasis remain unclear, nerves are regarded as an independent route of metastasis, in addition to the vascular and lymphatic pathways. PNI is an important risk factor for more aggressive tumor characteristics and worse outcomes in various tumors, such as gastric cancer (GC) (1–4). Moreover, accumulating evidence has demonstrated that PNI may predict treatment response in patients with GC (3, 5). Therefore, accurate detection of PNI is vital for personalized therapeutic strategies. Currently, the PNI status can only be confirmed postoperatively. Although biopsy may help determine the PNI status preoperatively, biopsy samples cannot reflect the whole landscape of the tumor, which leads to a risk of false negatives. Conventional radiological approaches, including magnetic resonance imaging and computed tomography (CT), fail to identify PNI status (6, 7). Thus, effective methods to determine the PNI status in GC patients preoperatively are urgently needed.

Radiomics techniques offer new insights into image data processing and can translate images into mineable data, allowing the detection of microscopic characteristics and heterogeneity of tumors that are indistinguishable by naked eye from conventional CT images (8). Accumulating evidence has shown the application potential of radiomics in multiple fields, including differential diagnosis, predicting metastasis and treatment efficacy (9–11). Moreover, the radiomics approach can be used as a noninvasive imaging biomarker to evaluate the characteristics of tumor microenvironment.

The predictive value of radiomics-based techniques for PNI has been widely studied in various types of cancer (12–15). However, radiomic studies focusing on PNI in GC are limited, and the sample sizes are relatively small (16). Therefore, the purpose of this study was to determine the value of the radiomics approach and clinicopathological factors for the prediction of PNI status based on a larger cohort of patients with GC.

## Materials and methods

### Patient enrollment

**Figure 1:**
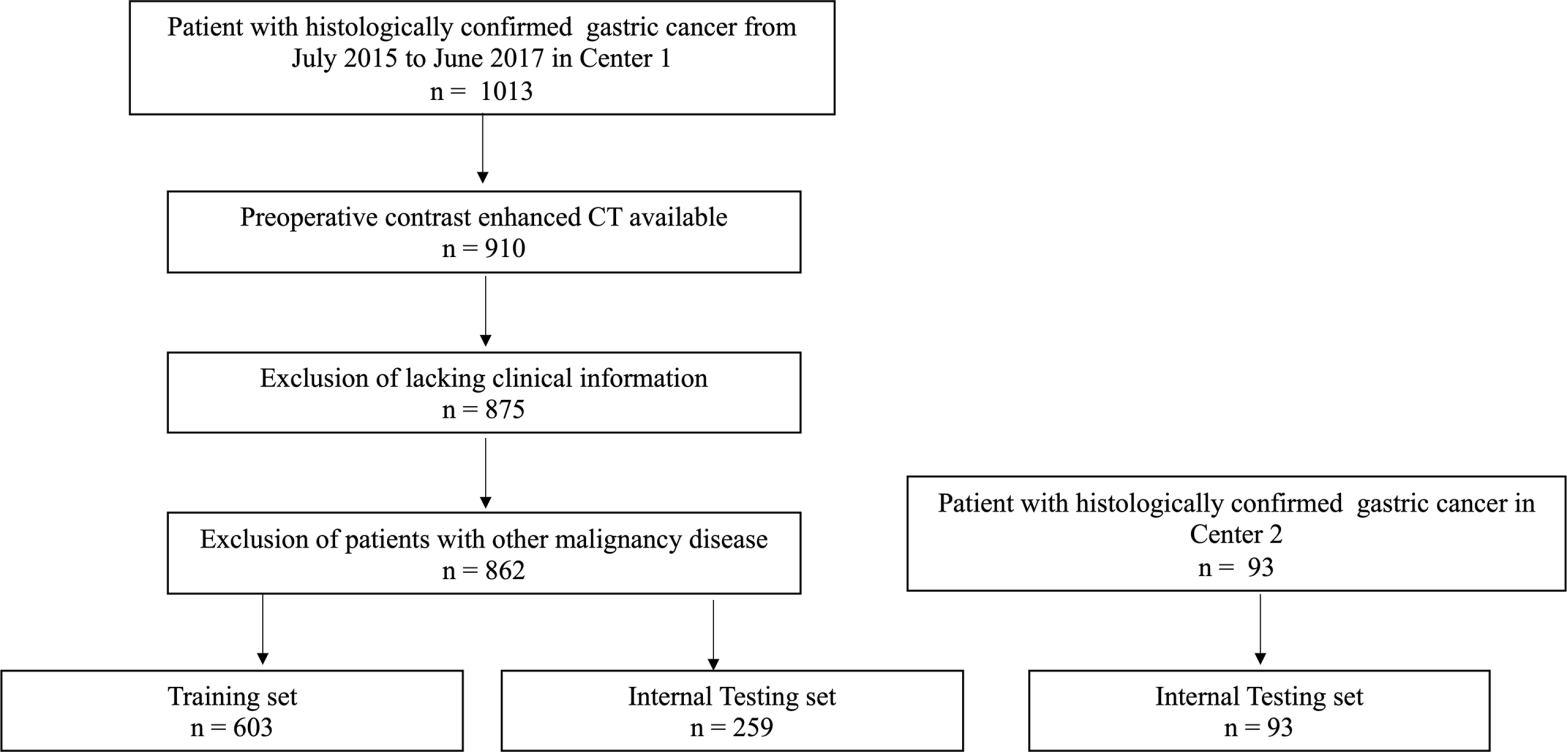
Recruitment pathways for patients.

Between July 2015 to June 2017, 862 consecutive GC patients were enrolled from in center 1, who were dividing them into the training (n=603) and internal testing groups (n=259) at a ratio of 7:3 randomly. Ninety-three patients were enrolled in center 2 as an external testing set. The inclusion criteria (1): received radical gastrectomy and D2 lymphadenectomy (2); pathologically confirmed GC (3); underwent abdominal contrast-enhanced computed tomography (CECT) scans two weeks before surgery (4); PNI status available; and (5) imaging quality met the following criteria: a) sufficiently distended gastric cavity and b) images devoid of significant artifacts. The exclusion criteria (1): lack of complete clinical records (2), received any treatment at the time leading up to the CT scan, and (3) suffered from other malignant diseases. Test results for cancer antigen (CA)72-4, CA199, carcinoembryonic antigen (CEA), and CA24-2 were also obtained. The threshold value of CA19-9, CA242, CA72-4, and CEA were 37 U/mL, 20 U/mL, 6.9 U/mL, and 5.0 μg/mL, respectively (17). Pathologic staging was assigned to each patient in accordance with the 8th edition of the AJCC staging manual. **Figure 1** shows the process of patient recruitment.

This study was approved by the ethical review board of our institution, and the requirement of obtaining informed consent was waived.

### CT image acquisition protocol

Abdominal CECT was performed using GE Discovery CT750 HD or Siemens Somatom Definition Drive scanner. Prior to the examination, patients were given oral doses of water to distend the stomach. CT scans were performed with standard setting: tube voltage of 120 kVp, auto tube current, and matrix of 512 × 512. The images were reconstructed with a section thickness of 1.25- or 1.5-mm. The arterial phase (AP), portal phase (PP), and delay phase (DP) images were obtained after delays of 20-30, 60 and 120 seconds, respectively.

### Lesion segmentation and feature extraction

Three phases of the abdominal CECT scans were analyzed. CT images were resampled into 1.0× 1.0 × 1.0 mm^3^ resolution using linear interpolation. To standardize the intensity range across scanners, Z-score normalization was utilized. Manually segmentation of the volume of interest (VOI) was performed with the agreement of two radiologists (with 7–10 years of abdominal CT imaging experience) by using 3D Slicer software (5.0.3). PyRadiomics package 2.2.0 was used for the feature extraction, extracting 1316 features from original and filtered images. Details of the radiomic features and pre-processing procedure are described at supplementary methods and https://pyradiomics.readthedocs.io/en/latest/.

### Radiomics signature establishment

To guarantee the robust and avoid overfitting of the chosen features, we used a multi-step dimensionality reduction method for feature screening. Firstly, intra- and inter-observer accessions were evaluated with intra- and inter-class correlation coefficients (ICCs). From the training set, 100 patients were chosen randomly, and two readers independently performed the VOI segmentation. When the values of ICCs exceed 0.85, the features were considered stable. Two weeks later, Reader 1 performed the segmentation again. When the values of ICCs exceed 0.85, the features were considered stable. Secondly, Spearman’s rank correlation coefficient was used to calculate the correlation between features, and one of the features with correlation coefficient greater than 0.9 between any two features is retained. Moreover, to decrease the redundancy of features to the greatest extent, the correlation between radiomics features and clinicopathological parameters were also analyzed. Only features with correlation coefficient less than 0.5 were retained. Thirdly, by using the Mann–Whitney U test, features that showed substantial variation between PNI+ and PNI- groups were selected. Finally, the retained features were inputted into least absolute shrinkage and selection operator (LASSO) for the determination of the features with best predictive ability.

After Lasso feature screening, six more machine learning (ML) algorithms including k-nearest neighbor (KNN), random forest (RF), support vector machine (SVM), decision tree (DT), eXtreme gradient boosting (XGBoost) and naïve Bayes (NB) were also applied for model training. We adopt 5-fold cross verification to obtain the final signature. Area under the receiver operating characteristic curve (AUC) was calculated to evaluate the diagnostic efficacy of the algorithms. The classifier with best AUC was chosen for radiomics signature establishment. Radiomics scores (R-scores) were calculated using the radiomics signature formula for each patient. To examine the importance of the selected features in the radiomics signature, the Shapley Additive explanations (SHAP) method was applied with best classifiers. The SHAP method is an approach for interpreting predictions of ML models and an extension of Shapley values, indicating the average marginal contribution of each feature over all combinations of features (18).

To eliminate the side effect of class imbalance on the model construction, the synthetic minority oversampling technique (SMOTE) was applied (19). The ML analysis was performed on the SMOTE-training set. In order to validation the performance of the radiomics signature in a real clinical environment, SMOTE was not performed in the test cohorts.

### Model establishment and evaluation

**Figure 2:**
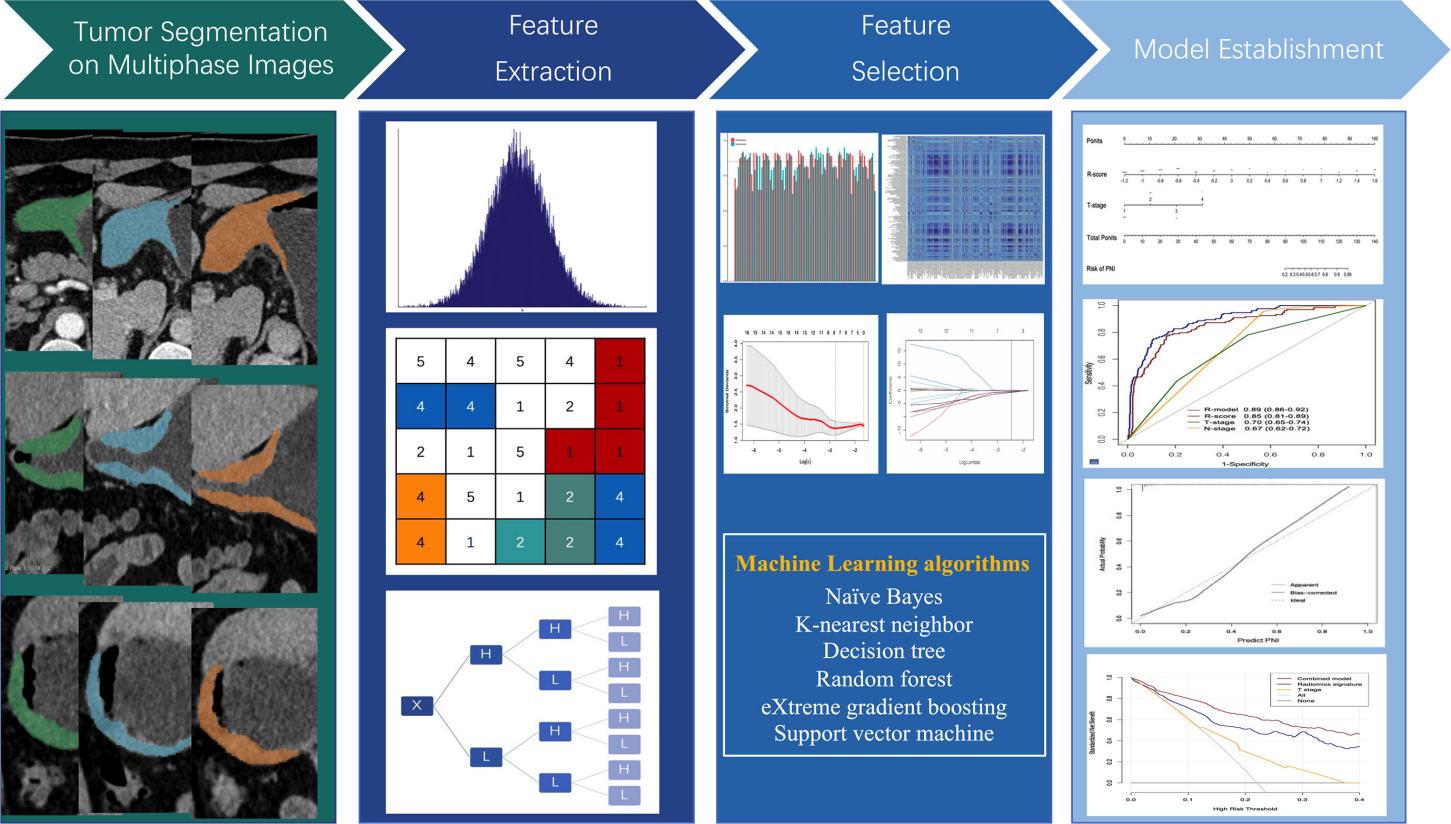
Flowchart of study design.

Multivariable logistic regression analysis was employed to select independent PNI risk factors. A combined model was constructed with radiomics signature and important clinicopathological parameters and represented as a nomogram. **Figure 2** shows the workflow of radiomics analysis.

### Statistical analysis

The chi-squared or Fisher’s exact test was used to compare categorical variable differences; while for continuous variables, the Mann–Whitney test was utilized. The degree to which different observers accurately reproduced lesion segmentation was determined by using the Dice similarity coefficient (DSC). A receiver operating characteristic (ROC) curve was applied to determine the radiomic signature discrimination power. The best cutoff threshold of the R-score was found to classify patients into low- and high-risk PNI groups using Maximized Youden index. Nomogram calibration was determined using the Hosmer–Lemeshow test. Decision curve analysis (DCA) was mapped out to assess the clinical utility of predictive models. R software (version 3.4.2) was utilized for statistical analyses.

## Results

### Clinical information

The training set consisted of 133 (22.1%) PNI+ patients, whereas the internal and external testing sets consisted of 59 (22.8%) and 34 (36.6%) PNI+ patients, respectively. In all three sets, the PNI positivity rate significantly correlated with higher T and N stages and poor differentiation status. The PNI+ and PNI- groups exhibited no significant variances regarding age, sex, tumor site, and serum biomarkers. **Table 1** shows the clinicopathological features of patients.

**Table 1:** Characteristics of the study population.

Variable	un-SMOTE Training Set (n=603)				Internal Testing Set (n=259)				External Testing Set (n=93)		
	PNI-(n=470)	PNI+(n=133)	*P*		PNI-(n=200)		PNI+(n=59)	*P*	PNI-(n=59)	PNI+(n=34)	*P*
Age			0.45					0.44			0.76
< 60	226 (48.1)	59 (44.4)			97 (48.5)		32 (54.5)		28 (47.5)	15 (44.1)	
≥ 60	244 (51.9)	74 (55.6)			103 (51.5)		27 (45.8)		31 (52.5)	19 (55.9)	
Gender			0.39					0.94			0.52
Male	310 (66.0)	93 (69.9)			138 (69.0)		41 (69.5)		36 (61.0)	23 (67.6)	
Female	160 (34.0)	40 (30.1)			62 (31.0)		18 (30.5)		23 (39.0)	11 (32.4)	
Tumor Site Upper Middle Lower Overlap	72 (15.3) 37 (7.9) 203 (43.2) 158 (33.6)	11 (8.3) 11 (8.3) 63 (47.4) 48 (36.1)	0.23		25 (12.5) 11 (5.5) 86 (43.0) 78 (39.0)		6 (10.2) 4 (6.8) 25 (42.4) 24 (40.7)	0.95	6 (10.2) 7 (11.9) 26 (44.1) 20 (33.9)	4 (11.8) 5 (14.7) 14 (41.2) 11 (32.4)	0.97
Pathologic T stage			<0.01					<0.01			0.03
T1 T2 T3 T4	118 (25.1) 67 (14.3) 17 (3.6) 268 (57.0)	1 (0.8) 3 (2.3) 2 (1.5) 127 (95.5)			68 (34.0) 44 (58.4) 10 (5.0) 78 (39.0)		2 (3.4) 2 (3.4) 1 (1.7) 54 (91.5)		11 (18.6) 9 (15.3) 11 (18.6) 28 (47.5)	1 (2.9) 2 (5.9) 5 (14.7) 26 (76.5)	
Pathologic N stage N0 N1 N2 N3	232 (49.4) 71 (15.1) 70 (14.9) 97 (20.6)	29 (21.8) 23 (17.3) 23 (17.3) 58 (43.6)	<0.01		105 (52.5) 31 (15.5) 21 (10.5) 43 (21.5)		11 (18.6) 4 (6.8) 15 (25.4) 29 (49.2)	<0.01	24 (40.7) 8 (13.6) 9 (15.3) 18 (30.5)	4 (20.6) 3 (8.8) 6 (17.6) 21 (61.8)	<0.01
Differentiation Well-Moderate Poor	81 (17.2) 389 (82.8)	7 (5.3) 126 (94.7)	<0.01		50 (25.0) 150 (75.0)		5 (8.5) 54 (91.5)	0.01	21 (35.6) 38 (64.4)	5 (14.7) 29 (85.3)	0.03
CEA ≥ 5.0 μg/mL < 5.0 μg/mL	81 (17.2) 389 (82.8)	20 (15.0) 113 (85.0)	0.55		26 (13.0) 174 (87.0)		13 (22.0) 46 (78.0)	0.54	–	–	–
CA19-9 ≥ 27 U/mL < 27 U/mL	80 (17.0) 390 (83.0)	27 (20.3) 106 (79.7)	0.38		27 (13.5) 173 (86.5)		14 (23.7) 45 (76.3)	0.06	–	–	–
CA242 ≥ 20 U/mL < 20 U/mL	58 (12.3) 412 (87.7)	11 (8.3) 122 (91.7)	0.19		14 (7.0) 186 (93.0)		8 (13.5) 51 (86.4)	0.11	–	–	–
CA72-4 ≥ 6.9 U/mL < 6.9 U/mL	88 (18.7) 382 (81.3)	17 (12.8) 116 (87.2)	0.11		28 (14.0) 172 (86.0)		12 (20.3) 47 (79.7)	0.24	–	–	–

Synthetic minority oversampling technique.

### Segmentation reproducibility

The DSC of the inter-observer segmentation was 0.91, indicating that the readers had a beneficial agreement.

### Radiomics signature establishment

**Figure 3:**
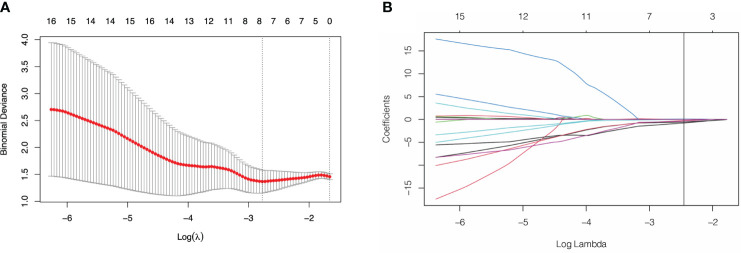
Feature selection using least absolute shrinkage and selection operator (LASSO) logistic regression. **(A)** Selection of tuning parameter (λ) in the LASSO model *via* 10-fold cross-testing based on minimum criteria. The AUC curve was plotted against log (λ). Dotted vertical lines were drawn at the optimal values by using the minimum criteria and the 1 standard error of the minimum criteria (the 1- standard error criteria). **(B)** LASSO coefficient profiles of the selected features. A vertical line was plotted at the optimal λ value, which resulted in 8 features with nonzero coefficients.

Of the 3948 features extracted from the VOIs in the training set, 2532 features were excluded because their ICCs were less than 0.85. Correlation coefficients of the retained 1416 features were calculated and 368 highly redundancy features were excluded. Then, 78 features that closely correlated with clinicopathological parameters were also excluded. Correlation matrix of radiomics features were shown in **Figure S1**. Between the PNI- and PNI+ groups, 46 of the retained 970 features that differed significantly were identified and added into LASSO analysis. Eight nonzero coefficient features including 1 feature from AP, 6 features from PP and 1 feature from DP were chosen (**Figure 3**). Subsequently, six MS algorithms including SVM, RF, DT, KNN, NB and XGBoost were also used to find the best classifier for establishment of signature. As shown in **Table 2**, the AUCs of LASSO, SVM, RF, DT, KNN, NB and XGBoost models were 0.85, 0.82, 0.65, 0.81, 0.75. 0.79 and 0.77 respectively. Therefore, LASSO algorithm was chosen for the radiomics signature construction. The R-score for each patient was computed using the formula shown in **Supplementary Data**. The distribution of the R-scores shows in **Figure S2**.

**Table 2:** Predictive performances of different machine learning classifiers.

Model	AUC	Accuracy	Sensitivity	Specificity
LASSO	0.85	0.82	0.77	0.83
SVM	0.82	0.79	0.74	0.80
RF	0.65	0.60	0.50	0.63
DT	0.81	0.78	0.70	0.80
KNN	0.75	0.71	0.74	0.75
NB	0.79	0.74	0.62	0.77
XGBoost	0.77	0.71	0.66	0.72

LASSO, least absolute shrinkage and selection operator; SVM, support vector machine; RF, random forest; DT, decision tress; KNN, k-nearest neighbor; NB, naïve Bayes; XGBoost, eXtreme Gradient Boosting; AUC, area under the receiver operating characteristic.

### Evaluation of radiomics signature’s predictive performance

**Figure 4:**
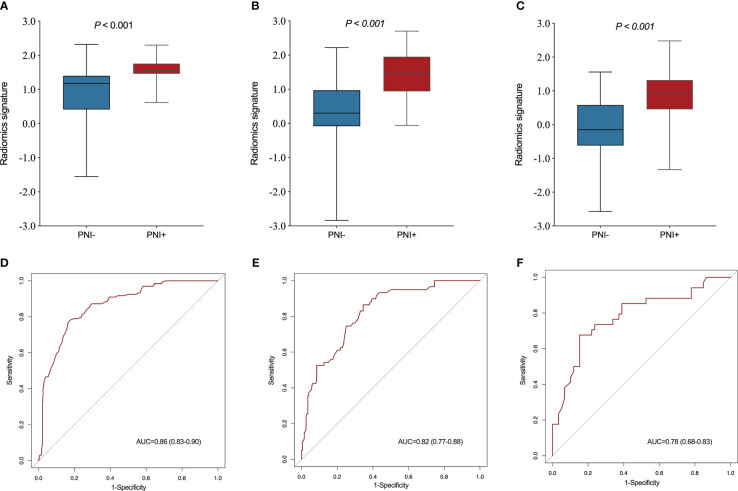
Comparison of radiomics score between perineural invasion (PNI) - and PNI + groups in the training **(A)**, internal testing **(B)** and external testing **(C)** sets. The ROC curves of the radiomics signature in the training **(D)**, internal testing **(E)** and external testing **(F)** sets.

A significant variation was showed in the R-score between patients with PNI and without PNI in the SMOTE-training (*P* < 0.001, **Figure 4A**), internal testing (*P* < 0.001, **Figure 4B**), and external testing (*P* < 0.001, **Figure 4C**) sets. The R-score showed good performance with an AUC of 0.86 (95% confidence interval (CI): 0.83–0.90) in the SMOTE-training (**Figure 4D**), 0.82 (95% CI: 0.77–0.88) in the internal testing (**Figure 4E**) and 0.78 (95% CI: 0.68–0.83) in the external testing sets (**Figure 4F**).

To evaluate feature importance and to improve the explainability of the radiomics signature, the SHAP values of the selected feature for each prediction were computed and visualized in the training set. A positive SHAP value indicated a high likelihood of a detection of PNI (**Figure S3**).

### Establishment and confirmation of nomograms

**Figure 5:**
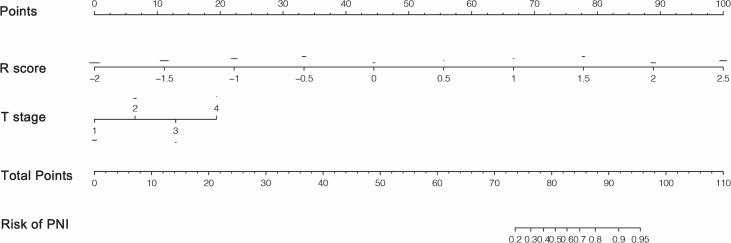
Radiomics nomogram based on radiomics score and clinicopathological factors.

The relationship between PNI, R-score, and clinicopathological parameters was evaluated using uni- and multivariable logistic regression analyses. As shown in **Table 3**, radiomics signature, T stage, and N stage were independent risk factors for PNI. With the advancement and application of endoscopic ultrasonography (EUS), the accuracy of preoperative T staging has largely improved using EUS combined with biopsy and conventional CT. However, accurate staging of lymph node metastasis (LNM) can only be assessed after surgery. Therefore, we constructed a combined model incorporating the R-score and T stages. This model was demonstrated as a nomogram for clinical use (**Figure 5**).

**Table 3:** Univariate and multivariate analyses of predictors of perineural invasion in gastric cancer .

Variable	Univariate analysis		Multivariate analysis	
	OR (95% CI)	P value	OR (95% CI)	P value
Gender (male vs. female)	0.83 (0.55-1.26)	0.39		
AGE (<60 vs. ≥ 60)	1.16 (0.79-1.71)	0.45		
Tumor site	1.19 (0.97-1.46)	0.10		
Differentiation	3.75 (1.69-8.32)	<0.01	2.29 (0.99-5.30)	0.06
T stage	3.56 (2.33-5.43)	<0.01	3.06 (2.00-4.68)	<0.01
N stage	1.63 (1.39-1.91)	<0.01	1.24 (1.04-1.47)	0.02
CEA	0.85 (0.50-1.45)	0.55		
CA 242	0.64 (0.33-1.25)	0.20		
CA 19-9	1.24 (0.76-2.01)	0.38		
CA 72-4	0.64 (0.36-1.11)	0.11		
Radiomics signature	7.81 (3.53-17.22)	<0.001	6.36 (3.01-16.06)	<0.01

CI, confidence interval.

**Figure 6:**
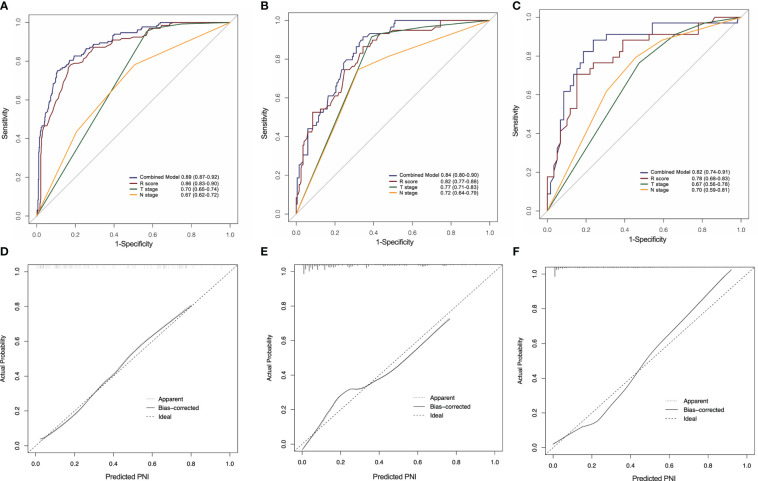
ROC curves of the radiomics nomogram for the prediction of PNI status in the training **(A)**, internal testing **(B)** and external testing **(C)** sets. Calibration curves of the nomogram in the training **(D)**, internal testing **(E)** and external testing **(F)** sets.

As shown in **Figure 6**, the combined model had an AUC of 0.89 (95% CI: 0.87–0.92) in the SMOTE-training set (**Figure 6A**), 0.84 (95% CI: 0.80–0.90) in the internal testing set (**Figure 6B**) and 0.82 (95% CI: 0.74–0.91) in the external testing set (**Figure 6C**). In all three sets, the nomogram outperformed both the radiomics signature and T-staging. The calibration curve matched well the actual and estimated values of the nomogram in all three sets (SMOTE-training: *P* = 0.86, **Figure 6D**; internal testing: *P* = 0.75, **Figure 6E**; and external testing: *P* = 0.80 **Figure 6F**). The distribution of the combined model scores shows in **Figure S2**. As shown in **Figure S4**, the DCA also confirmed that the combined model offers more benefit than radiomics signature and T stage.

## Discussion

This study established and validated a radiomic signature for predicting PNI in GC from multi-phase CECT images. The signature demonstrated a strong capacity for identifying PNI in all three sets. Furthermore, a combined model was constructed by incorporating the R-score with T stage. The model showed better discriminatory power than the radiomic signature and pathological factors.

Based on Japanese gastric cancer treatment guidelines, patients without LNM should receive endoscopic treatment rather than D2 lymphadenectomy (20). However, the PNI is an upstaging and poor outcome factor in N0 patients with GC (4). Endoscopic resection for early-stage GC with PNI may delay the early management of patients. In line with previous studies, our results revealed that the PNI rate significantly increased at higher T and N stages, indicating a stronger invasive ability of tumor cells and a higher risk of developing progressive disease. Furthermore, the PNI is predictor of the benefits of neo/postoperative chemotherapy or radiotherapy for GC (5). Therefore, precise preoperative evaluation of PNI plays a crucial role in precision and personalized treatment planning for GC.

Several studies have demonstrated the value of radiomics methods for identifying PNI based on different types of imaging devices. Huang et al. developed radiomics model based on CECT images which may be useful to detect PNI in colorectal cancer (12). Zhang et al. extracted radiomics features from T2 and diffusion-weighted MRI images and proposed a multiparametric clinical-radiomics model for prediction PNI in colorectal cancer (13). Ma et al. showed the predictive value of PET-CT based radiomics nomogram for PNI and prognosis in colorectal cancer (15). Zheng et al. previously reported a radiomics approach for prediction of PNI in GC. However, their study only included 154 patients from single institute. Additionally, the radiomics features were extracted solely from one phase of images. Liu et al. demonstrated that CT texture attributes are correlated with Lauren’s classification, vascular invasion status, and the differentiation degree of GC (21). Nevertheless, their feature analysis was based on the 2D maximum dimension and failed to predict the PNI status (21). Compared to previous studies, our study had clear advantages. In our study, a total of 955 GC patients (225 PNI+) were enrolled. The model was built based on a large cohort of GC patients, and further validated in independent sets of patients from two centers. Moreover, the radiomics features of our model were collected from 3D VOI of three-phase CECT images, enabling a comprehensive representation of lesion. The analysis process of our study increased the robust and reliability of our model.

The PNI is an important component of the tumor microenvironment (1). Complex interactions between neural, tumor, and stromal cells promote the development of PNI (22). The radiomics method has been extensively utilized in tumor microenvironment heterogeneity prediction (23). By analyzing high-dimensional information rather than simple semantic features, radiomic methods allow for comprehensive characterization of the tumor microenvironment (24). The radiomics features chosen in our signature were suggested to be useful for the characterization of intratumoral heterogeneity and clinical information. A previous systematic review reported that first order features were most reproducible radiomics features for characterization of tumor heterogeneity (25). In our radiomics signature, two first order features (entropy and uniformity) were selected. According to previous study, uniformity and entropy were the important feature for predicting prognosis of various malignancies (26, 27). Gray Level Dependence Matrix (GLDM) quantifies gray level dependencies which is defined as the number of connected voxels. Gray Level Run Length Matrix (GLRLM) features describes gray level runs, which are defined as the length in number of pixels, of consecutive pixels that have the same gray level value. Non-uniformity, which is one of the most important features in GLRLM and GLDM, was sleeted in our signature. The higher value of uniformity correlates with a greater heterogeneity (28). Neighbouring Gray Tone Difference Matrix (NGTDM) features quantifies the difference between a gray value and the average gray value of its neighbors. Contrast feature of NGTDM measures the spatial intensity change. High value of contrast indicates an image that exhibits large changes between voxels and their neighbourhood., which also suggest more heterogeneity in the texture patterns. Yang et al. reported that tumor size was positively associated with PNI (29). Nonetheless, since the precise confirmation of tumor size can only take place postoperative, this factor was not used in our model building. Nevertheless, a maximum 3D diameter, which was an indicator of tumor size, was determined as a key feature in our radiomics signature. Therefore, our radiomics signature offers promising insights into tumor microenvironment with good explainability for the prediction of PNI status.

Traditional serum tumor biomarkers are used commonly for the diagnosis and prognosis of GC (17, 30). Previous studies have reported that CEA levels are associated with PNI risk in colorectal cancer (31). However, according to our results, four markers, CEA, CA72-4, CA19-9, and CA24-2, showed no correlation with PNI. This indicates that serum biomarkers have limited utility in the prediction of PNI in GC.

This study has some limitations. First, evaluating radiomics features may not be consistent among scanners and institutions because of differences in the parameters used. Second, 3D lesion segmentation based on multiphase images are computationally complex and time-consuming. Third, the molecular mechanism of PNI in GC still unclear. The molecular factors participating in the development of PNI may provide information on the prognosis and therapeutic response. Epithelial-mesenchymal transition (EMT) is a molecular subtype that shows the worst survival for GC patients (32). Ahmadi et al. reported that EMT contributes to the development of PNI (22). Multiple reports have demonstrated the value of the radiomics approach in assessing gene mutation status (33, 34). Consequently, future studies will require additional radiogenomics analyses that correlate radiomic features with molecular profiles. Lastly, as with any other retrospective study, the current analysis may have included a selection bias. Therefore, larger prospective multicenter investigations should be performed to verify the applicability of this model.

In conclusion, our study demonstrates that radiomics analysis of preoperative CT images can provide useful information for predicting the presence of PNI in GC patients. The high accuracy of our radiomics model suggest that it could potentially be used as a non-invasive tool to help identify patients at high risk of PNI. Future prospective studies with larger cohorts are needed to validate our findings and assess the clinical utility of our radiomics model.

## Data availability statement

The raw data supporting the conclusions of this article will be made available by the authors, without undue reservation.

## Ethics statement

The studies involving human participants were reviewed and approved by Tianjin Medical University Cancer Institute and Hospital. Written informed consent for participation was not required for this study in accordance with the national legislation and the institutional requirements.

## Author contributions

XG: Conceptualization, Methodology, Data curation, Formal analysis, Writing – original draft, review & editing, Funding acquisition. JC: Conceptualization, Methodology, Data curation, Formal analysis. LW: Data curation. QW: Data curation. TM: Writing – review & editing, Supervision, Funding acquisition. JY: Writing – review & editing, Supervision, Project administration. ZY: Conceptualization, Supervision, Project administration, Resources, Funding acquisition. All authors contributed to the article and approved the submitted version
