# Supplementary material for: The value of machine learning based radiomics model in preoperative detection of perineural invasion in gastric cancer: a two-center study
Source: Front Oncol. 2023 Jun 14;13:1205163. doi: 10.3389/fonc.2023.1205163 (PMC10303108; doi:10.3389/fonc.2023.1205163)
Supplement: Supplementary file 1 [file DataSheet_1.docx]

**I. Supplementary methods and data**

**1. Radiomic feature extraction and pre-processing**

Before feature extraction, CT images were resampled into 1.0× 1.0 × 1.0 mm^3^ resolution using linear interpolation. To standardize the intensity range across scanners, Z-score normalization was utilized. Radiomic features were extracted from the original images of tumors and images transformed by wavelet and Laplacian of Gaussian (LoG) filtration. Original images were wavelet filtered with three directions, resulting eight different combinations of feature. LoG filtration images were generated with sigma=1, 2, 3, 4 and 5 mm.

Features with more than 5% incomplete values (i.e., unexpected zeros and NA) were regarded as unstable and removed. Remainder of incompleteness were interpolated by their median feature values, outliers and extreme values were winsorized by nearest IQR respectively. Then, features were scaled into 0 to 1 by z-score transformation to ensure the comparability of the dynamic range of radiomic features before selection.

**2. Radiomics signature formula**

Radiomics score = 0.851+2.833×Wavelet-HLL-Firstorder-Entropy (AP)

+0.776 ×Wavelet-LHH- GLDM-NonUniformity (PP)

-0.232 × GLSZM-High gray level zone emphasis (PP)

+1.306 ×log-sigma-1.0mm-3D-GLDM-Gray Level Variance (PP)

-1.911 × Wavelet-HHH- GLRLM-RunEntropy (PP)

-0.068 × Wavelet-LLH-Firstorder-Uniformity (PP)

+0.858 × Wavelet-LHH-NGTDM-Contrast (PP)

+1.077 × Maximum 3D diameter (DP)

**II. Supplementary Figures**

**Figure S1**. Correlation matrix of radiomics features

**
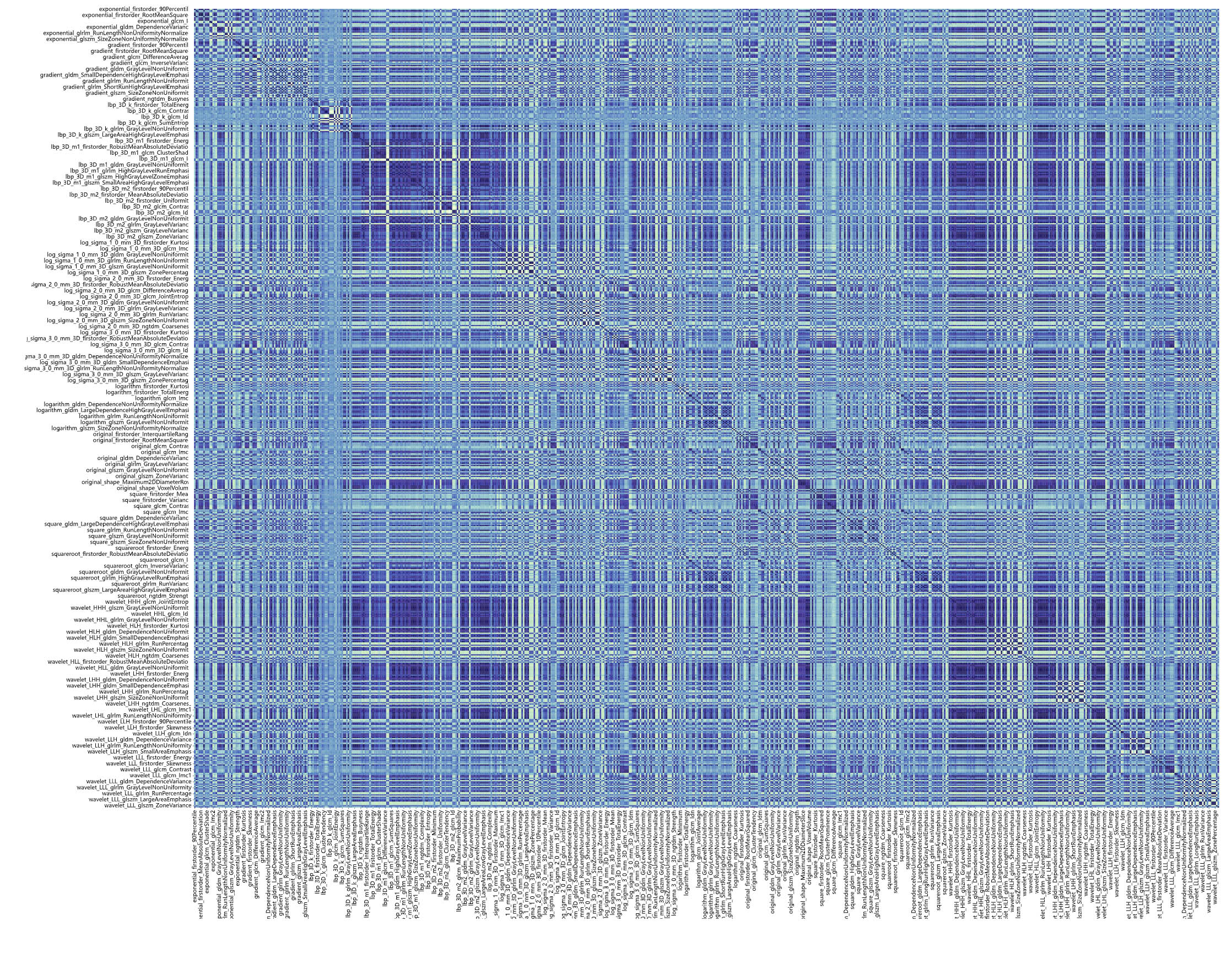
**

**Figure S2.** Radiomics score for each patient in the training (A), internal testing (B) and external testing (C) sets; Combined model score for each patient in the training (D), internal testing (E) and external testing (F) sets

**
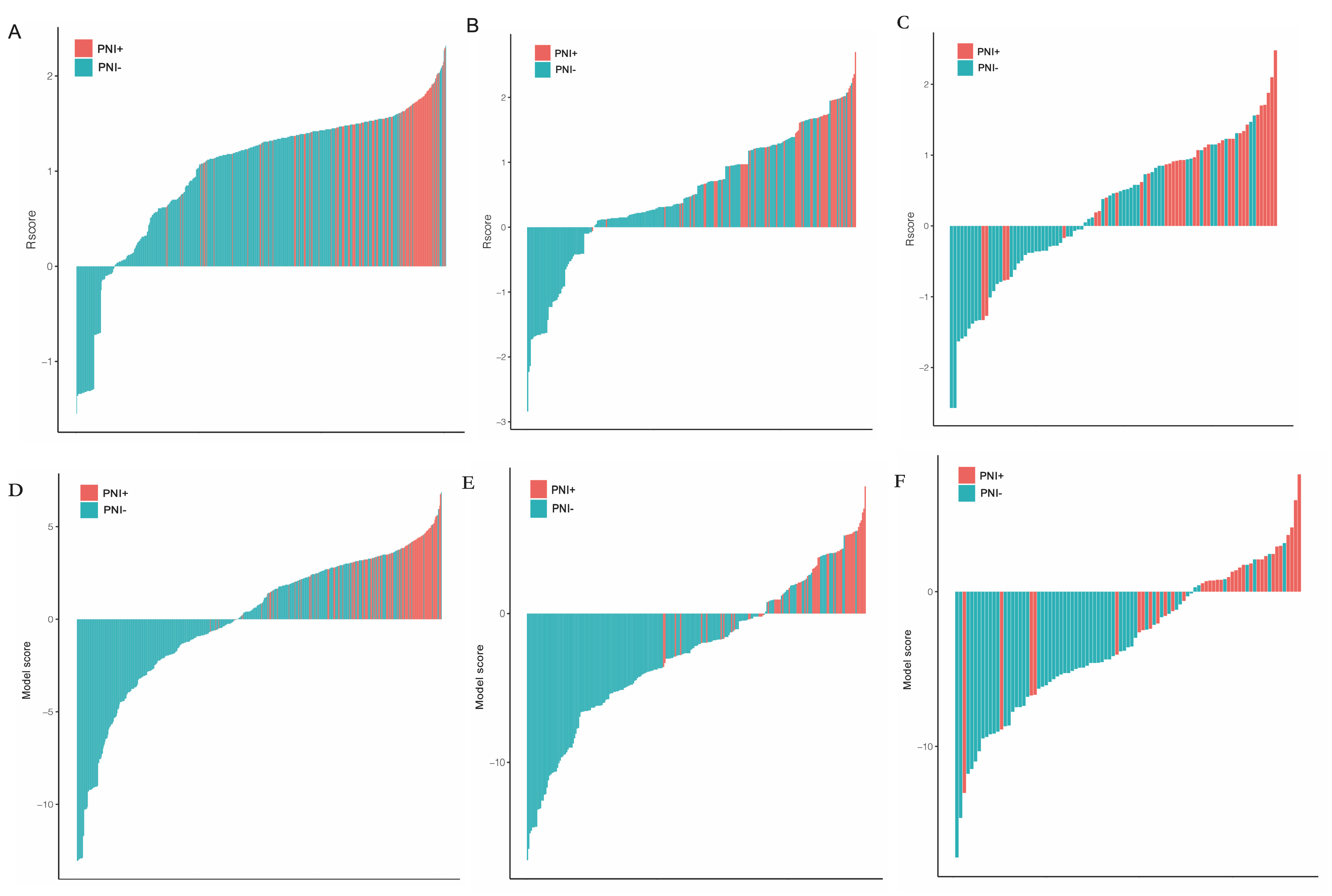
**

**Figure S3.** The Shapley additive explanations (SHAP) summary plot of features impact on predicted probability through colors, including positive and negative predictive effects.

**
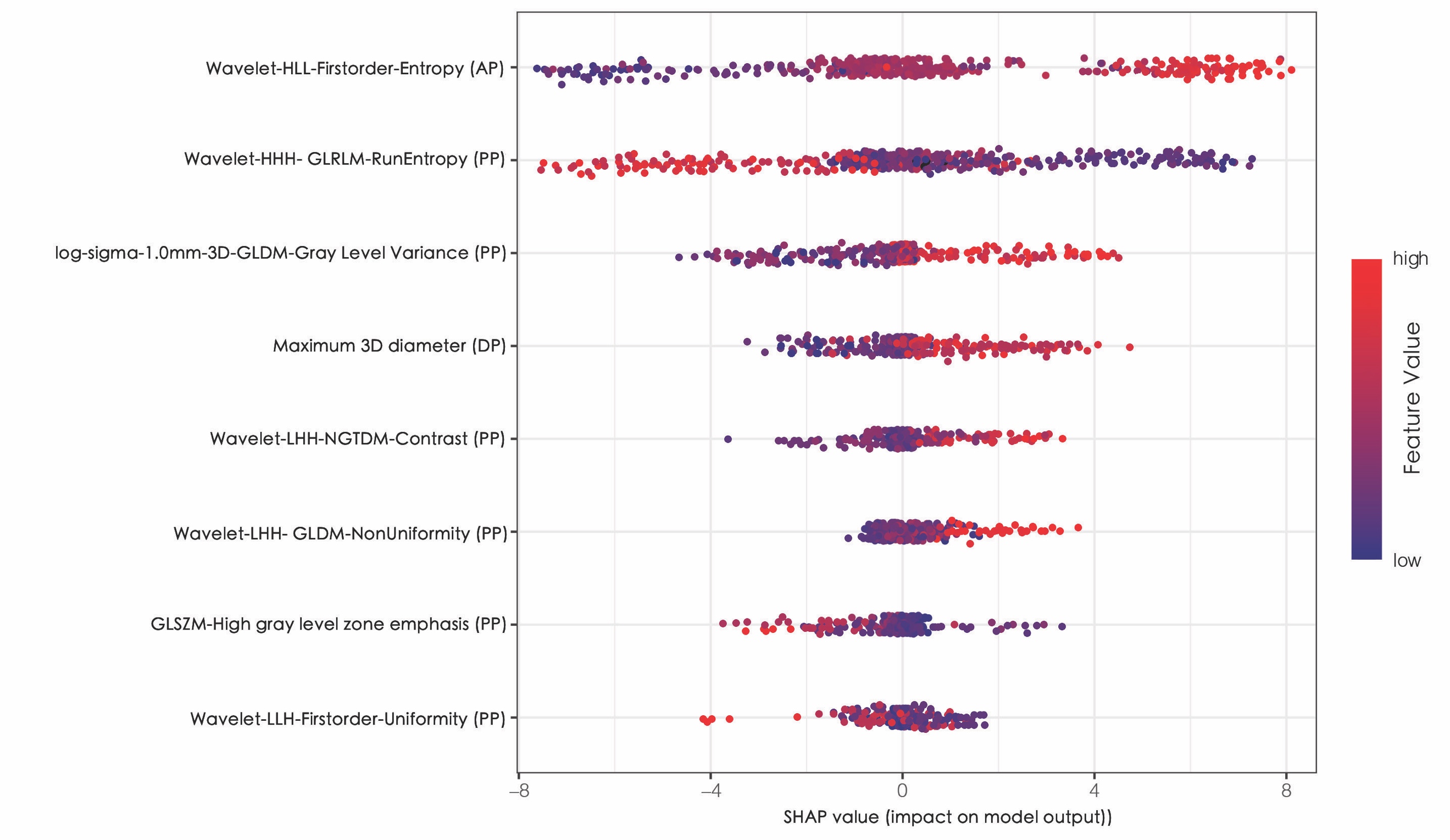
**

**Figure S4.** Decision curve analysis for radiomics signature, T stage and combined model. The Y-axis represented the net benefit. The red line represented the combined model. The blue line represented the radiomics signature. The orange line represented the T stage.
